# Effect of fish oil intake on glucose levels in rat prefrontal cortex, as measured by microdialysis

**DOI:** 10.1186/1476-511X-12-188

**Published:** 2013-12-26

**Authors:** Isy F de Sousa, Adriana P de Souza, Iracema S Andrade, Valter T Boldarine, Claúdia MO Nascimento, Lila M Oyama, Mônica M Telles, Eliane B Ribeiro

**Affiliations:** 1 Departamento de Fisiologia, Universidade Federal de São Paulo (Unifesp), Rua Botucatu, n° 862 – 2° andar, Vila Clementino, São Paulo, SP 04023-062, Brazil

**Keywords:** Food intake, Obesity, Long-chain omega-3 fatty acids, Brain microdialysis

## Abstract

**Background:**

Brain glucose sensing may contribute to energy homeostasis control. The prefrontal cortex (PFC) participates in the hedonic component of feeding control. As high-fat diets may disrupt energy homeostasis, we evaluated in male Wistar rats whether intake of high-fat fish-oil diet modified cortical glucose extracellular levels and the feeding induced by intracerebroventricular glucose or PFC glucoprivation.

**Methods:**

Glucose levels in PFC microdialysates were measured before and after a 30-min meal. Food intake was measured in animals receiving intracerebroventricular glucose followed, 30-min. later, by 2-deoxy-D-glucose injected into the PFC.

**Results:**

The fish-oil group showed normal body weight and serum insulin while fat pads weight and glucose levels were increased. Baseline PFC glucose and 30-min. carbohydrates intake were similar between the groups. Feeding-induced PFC glucose levels increased earlier and more pronouncedly in fish-oil than in control rats. Intracerebroventricular glucose inhibited feeding consistently in the control but not in the fish-oil group. Local PFC glucoprivation with 2-DG attenuated glucose-induced hypophagia.

**Conclusions:**

The present experiments have shown that, following food intake, more glucose reached the prefrontal cortex of the rats fed the high-fat fish-oil diet than of the rats fed the control diet. However, when administered directly into the lateral cerebral ventricle, glucose was able to consistently inhibit feeding only in the control rats. The findings indicate that, an impairment of glucose transport into the brain does not contribute to the disturbances induced by the high-fat fish-oil feeding.

## Background

The homeostatic control of energy homeostasis operates through peripheral signals integrated at the central nervous system (CNS), which modulates the activity of hypothalamic anabolic and catabolic neurons [1]. The long-term control depends on leptin and insulin signaling of body adiposity while the short-term control monitors hunger and satiety through signaling by gastrointestinal hormones [2].

Furthermore, the hedonic component of energy balance control exerts a pivotal modulation of the homeostatic influences on food intake [3,4]. Although of high physiological importance to feeding behavior, the reward properties of food may lead to excessive intake that surpasses the metabolic needs and leads to obesity. Mesocorticolimbic structures, including the prefrontal cortex (PFC), have been directly implicated in the hedonic aspect of feeding regulation [5-7].

Besides the mentioned hormones, the nutrients themselves may play a role as signals to the CNS. We and others have observed that central glucose administration reduced feeding in rats [8-12]. Glucose has been shown to modify the activity of both glucose-excited and glucose-inhibited neurons, whose existence has been demonstrated in the hypothalamus and also mesocorticolimbic structures [13,14]. Glucose signaling at these areas has been attributed a potential role not only as an acute satiety factor but also as a long-term feeding regulator [15].

High intake of fat-dense foods is a relevant factor in the obesity epidemic and its comorbidities [16]. The consumption of high-fat diets may affect the central mechanisms of feeding regulation as well as peripheral metabolism and the predominant fat type has been shown to be a relevant aspect conditioning the diet effects. Animal studies have found that saturated and n-6 and n-9 polyunsaturated fatty acids (PUFAs) are more involved in the genesis of insulin resistance, while n-3 PUFAs have been associated with less deleterious effects and even amelioration of the aspects affected by the other fats [17-21].

In rats, we have previously found that the long-term intake of trans-fat diet or n-6 PUFAs diet impaired hypothalamic insulin signaling and insulin hypophagia while n-3 PUFA diet had no such effects [22,23]. Additionally, n-6 PUFA diet caused neuronal activation of orexigenic hypothalamic sites while n-3 PUFA diet increased the activity of anorexigenic sites. On the other hand, the n-3 diet impaired the serotonergic activity at the hypothalamus [24,25].

The present work was aimed at evaluating whether the consumption of high-fat diet enriched with fish oil (source of n-3 PUFAs) modified PFC glucose extracellular levels as well as the feeding response to intracerebroventricular glucose or to PFC glucoprivation.

## Results

### Body weight gain, white fat mass and fasting serum glucose and insulin

No significant difference was observed between the groups on body weight (p = 0.3838) while white fat mass was higher in the fish-oil group than in the control group (p = 0.0005). The levels of glucose were significantly higher in fish-oil than in control rats (p = 0.016) while insulin levels were similar between the groups (Table 1).

**Table 1:** Body weight gain, fat depots weight, and serum glucose and insulin levels of control and fish-oil rats

	**Control**	**Fish-oil**
**Body weight (g)**	414 ± 9 (14)	401 ± 12 (14)
**†White fat (g/100 g)**	2.26 ± 0.14 (14)	3.39 ± 0.23* (14)
**Glucose (mg/dl)**	105.1 ± 3.7 (11)	130.8 ± 8.7* (12)
**Insulin (ng/ml)**	0.60 ± 0.07 (10)	0.52 ± 0.04 (9)

Values are expressed as means ± S.E.M.

†Sum of retroperitoneal, mesenteric and gonadal fat depots.

*p < 0.05 *vs.* Control. () number of animals.

### Effect of food intake on extracellular levels of glucose in PFC

For 30 minutes during microdialysates collection, the rats had access to their respective diets added with condensed milk, in the proportion of 1 g of diet to 1.5 g of condensed milk. Table 2 shows the composition of the condensed milk diets. The acute food mass ingestion was similar between the control (2.84 ± 0.40 g) and the fish-oil (2.64 ± 0.55 g) group. The intakes of carbohydrate, protein and fat, as well as energy intakes, were also similar between the groups. However, dietary fiber intake was lower in the fish-oil group in relation to the control group (p < 0.0001).

**Table 2:** Composition of the control and fish-oil diets available during the acute experiments

	**Treatment diets (%)**		**†Condensed milk diets (%)**		**‡30 min. food intake (g)**	
**Group**	**Control**	**Fish-oil**	**Control**	**Fish-oil**	**Control**	**Fish-oil**
**Kcal/100 g**	287.7	364.3	310.1	340.7	8.81 ± 1.24	8.99 ± 1.87
**Carbohydrate**	39.8	28.9	48.9	44.6	1.39 ± 0.20	1.18 ± 0.24
**Fat**	4.5	17.5	6.9	12.1	0.20 ± 0.03	0.32 ± 0.07
**Protein**	22	22.8	13.3	13.6	0.38 ± 0.05	0.36 ± 0.07
**Dietary Fiber**	13.8	3.7	5.5	1.5	0.16 ± 0.02	0.04 ± 0.01*

**†**Composition of the treatment diets blended with condensed milk (1:1.5; g:g).

**‡**n = 21 (control) and 14 (fish-oil).

Values are expressed as means ± S.E.M.; *p < 0.05 *vs.* Control.

Baseline values of glucose levels in the PFC were similar between control (217 ± 42 ng/10 μl) and fish-oil-group rats (166 ± 29 ng/10 μl, p = 0.38).

The chronic diet treatment had a significant interaction with sampling time after acute food intake (F_(10,330)_ = 2.195, p = 0.017). The PFC glucose levels were significantly increased in control animals at the times 60′, 90′, 120′, and 150′ after food presentation, with increments of 24% to 35%.

**Figure 1:**
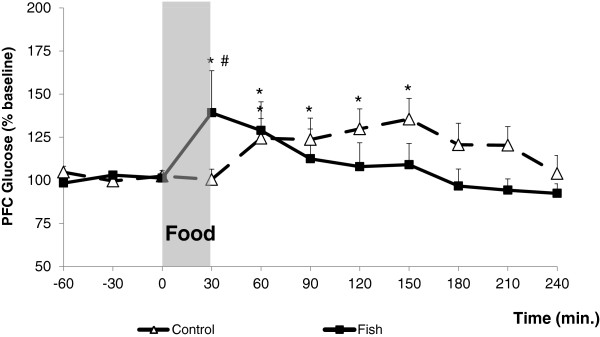
**Effect of acute food intake on extracellular levels of glucose in the prefrontal cortex.** Extracellular levels of glucose in the prefrontal cortex of control (N = 21) and fish-oil (N = 14) animals, before (baseline: -60, -30 and 0), during, and after food presentation. *p < 0.05 *vs.* baseline; #p < 0.05 *vs.* Control.

In the fish-oil group, the extracellular levels of glucose were significantly increased at 30 (39% increase) and 60 (28% increase) minutes after food presentation. The levels of glucose were significantly higher in the fish-oil group than in the control group at the time 30 minutes after food presentation (p = 0.014, Figure 1). The area under the curve of glucose levels over time was similar between control (8720 ± 1268) and fish-oil groups (8737 ± 1298, p = 0.993).

### Effects of intracerebroventicular glucose with or without intracortical 2-deoxy-D-glucose (2-DG) on food intake of control and fish-oil groups

**Figure 2:**
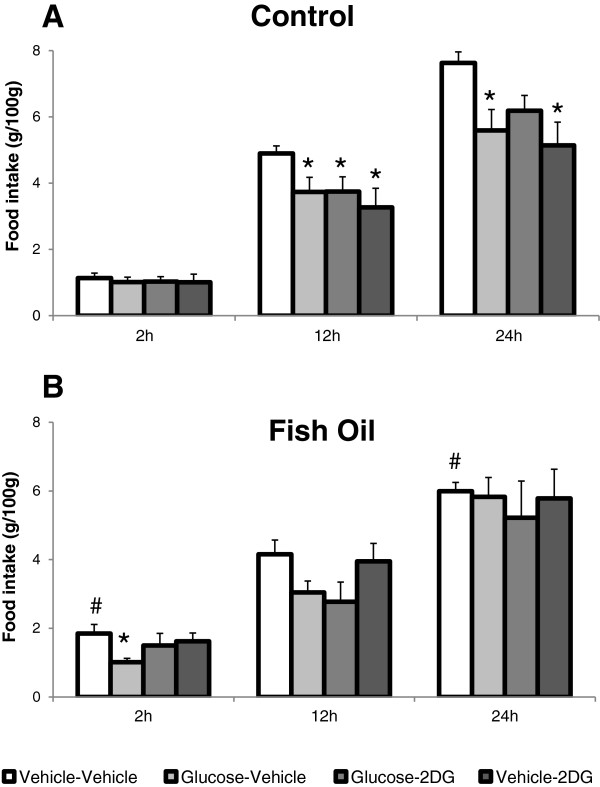
**Food intake of control and fish-oil groups after 2-DG, glucose or vehicle injections.** Food intake of control **(A)** and fish-oil **(B)** groups, 2, 12, and 24 hours after injections of vehicle-vehicle (N = 13-14 and 11–12), glucose-vehicle (N = 10-11 and 8–9), glucose-2DG (N = 11 and 9) or vehicle-2DG (N = 8 and 6–7). 2-DG or vehicle were injected in the PFC 30 min. after the i.c.v. injection of either glucose or vehicle. *p < 0.05 *vs*. vehicle-vehicle. #p < 0.05 *vs*. control.

Two hours after the injections, a significant diet effect (F_(3,69)_ = 6.573, p = 0.012) was detected. The fish-oil group injected with vehicle-vehicle had an ingestion significantly higher than that of the respective control group (p = 0.022, Figure 2). The injection of glucose-vehicle caused a small but significant inhibition of 2-h ingestion (p = 0.008). No significant diet *versus* injection interaction was detected.

A significant injection effect was detected at time 12 hours (F_(3,70)_ = 4.036, p = 0.010). In the control group, food intake was significantly inhibited 12 hours after the i.c.v. injection of glucose (p = 0.039), and the intracortical injection of 2-DG, performed 30 minutes after i.c.v. glucose, failed to modify glucose-induced hypophagia (p = 0.041). The intracortical injection of 2-DG after i.c.v. vehicle also inhibited 12-h feeding (p = 0.009). No significant effects were observed in the fish-oil group. No significant diet *versus* injection interaction was detected.

Anova showed a significant effect of injections (F_(3,69)_ = 3.213, p = 0.054). The post- hoc test showed that the 24-h intake of the fish-oil group was lower than that of the control group (p = 0.036). In the control group, the i.c.v. injection of glucose promoted a significant inhibition of 24-h food intake (p = 0.009). The intracortical injection of 2-DG attenuated glucose-induced hypophagia, since intake was not significantly different from the vehicle-vehicle group (0.063). The intake was also significantly inhibited by the PFC injection of 2-DG after i.c.v. vehicle (p = 0.004). No significant injections effects were detected in the fish-oil group.

### Verification of dialysis membrane and intracortical cannula positioning

**Figure 3:**
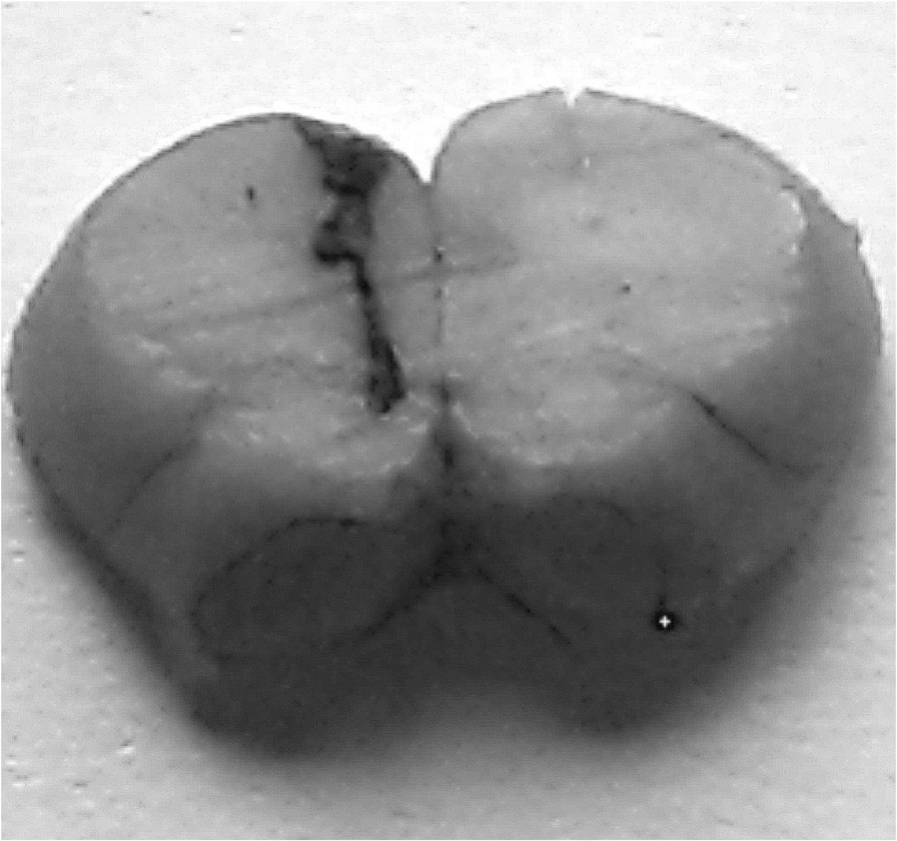
Localization of the microdialysis membrane in the prefrontal cortex.

Figure 3 shows a representative photograph of a brain section showing the positioning of the microdialysis membrane. Data analysis included only those rats in which the microdialysis membrane was correctly located in the PFC. The same verification was made for the placement of the guide cannula for intracortical injections (not shown).

## Discussion

In the present study, we observed that rats fed for 8 weeks with the fish-oil diet oil had increased fat pads weight with normal body weight. Normal body weight has been shown in mice fed n3-rich diet and ascribed to increased energy expenditure [26]. However, high fat oxidation and diminished expression of lipogenic factors have also been found [18,27], data not compatible with the increased fat pads weight observed in this study.

Increased expression of proopiomelanocortin (POMC) and reduced expression of neuropeptide Y (NPY) at the hypothalamus have been observed after fish-oil diets [28]. In our laboratory, we have described that the feeding-induced neuronal activation, as evidenced by c-Fos expression, occurred predominantly in the lateral portion of the arcuate nucleus, as well as the ventromedial hypothalamus (VMH), and paraventricular nucleus of hypothalamus (PVH), hypothalamic areas expressing anorexigenic neuropeptides [24]. These findings agree with the present observation of decreased daily food intake of the fish-oil rats.

Available data on the effects of n3-PUFA on body weight and composition in humans are controversial. Although it has been suggested that the beneficial effects of n-3 PUFAs are restricted to lean individuals [29], in overweight/obese subjects, n-3 PUFA supplementation improved the body weight loss induced by caloric restriction [30] while it improved the adipose mass reduction induced by exercise, with no effect on energy expenditure [31]. On the other hand, eicosapentaenoic acid/docosahexaenoic acid supplementation failed to modify the response of overweight individuals to nutritional/exercise intervention [32]. We have previously shown that n-3 PUFAs comprise 73% of the total fatty acids content of the fish-oil diet used in the present study, 39% of which is eicosapentaenoic acid and 24% is docosahexaenoic acid, while the control diet has only 7% as n-3 PUFAs [23].

The fish-oil group presented hyperglycemia with normal insulinemia, indicating insulin resistance. This contrasts with many studies that have associated the consumption of n-3 PUFAs to improved glycemic control and insulin sensitivity [33,34]. Diminished adipocytes size has been reported in obese/diabetic db/db mice after high-fat n-3 diet [35], what would have a positive effect on adipokines secretion and insulin sensitivity [36].

On the other hand, findings similar to the present ones, of insulin resistance after n-3 PUFAs treatment have been found in other studies in rodents [28] and humans [37,38]. In diabetic hypertensive humans, supplementation with eicosapentaenoic acid/docosahexaenoic acid induced fasting hyperglycemia [38,39] while hyperlipidemic humans showed increased insulinemia with normal glycemia [37].

We observed that the fasting baseline levels of glucose in PFC microdialysates were not affected by the long-term intake of the fish-oil diet. We then performed experiments to ascertain whether the intake of food modified the level of glucose recovered in the microdialysates. The diets consumed acutely had asimilar carbohydrates content although they differed in fat and fiber content.

The intake of food induced significant increases of PFC glucose in both the control and the fish-oil groups. Feeding-induced increase of glucose levels has also been reported in microdialysates of the ventromedial and the lateral hypothalamus of normal rats [40,41]. The fish-oil group showed a more pronounced increase and an earlier response, in comparison to the control group. This event could not be explained considering the carbohydrate content of the diet, since the same amount of this macronutrient was ingested by both groups during the 30-minutes meal. Although the control diet had higher fiber content than that of the fish-oil diet, the small amount of fiber ingested by both groups was probably not effective in producing a relevant difference in nutrients absorption. Furthermore, since the fish-oil group ate more fat than the control group, it is likely that the groups had similar carbohydrates absorption, as the fat content of the diet decreases glycemic index [42].

The present observations agree with the finding that an oral glucose overload increased striatum microdialysates glucose more pronouncedly in streptozotocin-diabetic than in control rats [43]. On the other hand, glucose flux to the parietal cortex has been found to be decreased in streptozotocin-diabetic rats, regardless the glycemic level [44]. Reduced glucose transport into the brain has also been reported in diabetic rats with normalization by glycemic control with chronic insulin [45].

Although we have not measured the feeding-induced glycemic levels, we observed fasting hyperglycemia in the fish-oil group, suggesting that the different PFC response to feeding between control and fish-oil groups could be related to their glycemic differences. There are findings supporting the view that glycemic levels influence the extracellular levels of glucose in the brain. For example, microdialysate glucose levels in the frontoparietal cortex of rats have been shown to be linked to the glycemic variations induced by venous glucose infusion [46]. Similar findings were obtained in the cingulate cortex, in which extracellular glucose, as measured by glucose-sensitive microelectrodes, paralleled the glycemic levels produced by intraperitoneal glucose [47]. In humans with severe brain injury, a positive correlation between glycemia and brain microdialysate levels of glucose has been reported in non-injured unlike injured areas [48]. On the other hand, findings pointing to the absence of a direct link between plasma and brain extracellular glucose levels have also been provided. In normal rats, such demonstration has been made for feeding-induced glucose levels in the hypothalamus [40] and glucose injection-induced extracellular levels in the hippocampus [49], as evaluated by microdialysis.

The finding that glucose increased efficiently in the PFC of the fish-oil group after feeding contrasts with the mild feeding inhibition by i.c.v. glucose in this group, in comparison to the control group.

Unlike the prefrontal cortex, the hypothalamus has been largely studied with respect to the mechanisms of glucose interaction with neurons. In this region, glucose-inhibited and glucose-excited neurons have been characterized, both types displaying abnormal response to glucose variations in obese rats. Particularly in the medial hypothalamus, increment of glucose levels has been shown to decrease firing rate of NPY-expressing glucose-inhibited neurons while increasing that of POMC-expressing neurons, thus leading to feeding inhibition [13,50-52]. Thus, failure of i.c.v. glucose to consistently inhibit feeding in the fish-oil group could rely on impaired glucose action on these neurons. This notion is compatible with the demonstration that glucose transporter 2 null mice became hyperphagic, had disrupted hypothalamic neuropeptides expression, and failed to respond with hypophagia to either i.c.v. or intraperitoneal glucose [53].

The PFC has been found to have neurons whose activity changed in response to glucose, the majority of which (69%) has been found to be glucose-inhibited, although glucose-excited neurons have also been identified. These neurons have been implied in the feeding control influences exerted by the prefrontal cortex [14]. Although their phenotype has not yet been ascertained, it is likely that these neurons participate in the mechanisms affecting the hedonic component food intake control. The present finding of attenuation of glucose-induced hypophagia by PFC glucoprivation indicates that local glucose metabolism may be relevant to the glucose effect. In this respect, it is noteworthy that we have observed, in a previous study in hyperglycemic rats fed saturated high-fat diet, exacerbated increase of glucose levels in the ventromedial hypothalamus and abolition of central glucose-induced hypophagia [54].

Besides glucose, fatty acids signaling in the central nervous system has been associated with inhibition of NPY neurons and decreased feeding and hepatic glucose production [55]. Importantly, consumption of high-fat diet has been shown to impair the ability of i.c.v. oleic acid to induce these effects [56]. It is possible to suggest that these defects induced by high-fat feeding may have impaired glucose effects as well. In humans, euglycemic or hyperglycemic conditions induced activation of medial PFC, what was associated with less interest in food stimuli, and that the PFC activation was impaired in obese individuals [57].

In the present study, the PFC injection of 2-DG inhibited 12-h and 24-h food intake in the control but not in the fish-oil group. This agrees with the demonstration of attenuation, in high fat-fed rats, of the hyperphagic response to glucoprivation induced by either systemic or IV ventricle injection of 5-thio-D-glucose [58].

Although generalized glucoprivation, as induced by either peripheral or i.c.v. 2-DG, is known to induce feeding, the response to localized injection is controversial, as absence of an effect and both feeding inhibition and stimulation have been reported after 2-DG injection into various brain sites [59,60]. The physiological significance of the present finding of feeding inhibition by 2-DG local PFC administration is not clear. However, the present concomitant observation of attenuation of this effect in the fish-oil group strongly suggests that glucose sensing is disrupted in this situation. Additional experiments are warranted to elucidate the part played by glucose metabolism in this brain site and the mechanisms affected by fish oil consumption.

## Conclusions

The present experiments have shown that, following food intake, more glucose reached the prefrontal cortex of the rats fed the high-fat fish-oil diet than of the rats fed the control diet. However, when administered directly into the lateral cerebral ventricle, glucose was able to consistently inhibit feeding only in the control rats. The findings indicate that, an impairment of glucose transport into the brain does not contribute to the disturbances induced by the high-fat fish-oil feeding.

## Methods

### Animals and diets

All animal experiments were performed in accordance with the directives of the Brazilian Council on Animal Research and the Committee on Animal Research Ethics of the Federal University of Sao Paulo. Male Wistar rats were maintained under controlled conditions of lighting (12:12 h light–dark cycle, lights on at 6 am) and temperature (24 ± 1°C). At two-months of age, the rats were randomly assigned to receive standard chow diet (15% energy from fat; Nuvilab CR1, Nuvital, PR, Brazil) or high-fat diet prepared by adding 20% (w/w) of fish oil (ROPUFA® ‘75′ω-3, Roche, DSM Nutritional Products, Brazil) to the control diet (fish-oil group: 52% energy from fat). The high-fat diet also contained (w/w) 10% sucrose and 20% casein, to obtain a protein/energy ratio similar to that of the control diet. The composition of the treatment diets is shown in Table 2. The fatty acids composition of the diets has been previously published [23]. The diet treatments were applied for 8 weeks.

### Placement of intracerebroventricular and PFC cannulas

After the 8 weeks on the diet treatments, the rats were anaesthetized (ketamine/xylazine 66.6/13.3 mg/Kg, i.p.) for microdialysis and intracortical guide cannulas introduction. They were stereotaxically implanted with 21-gauge guide cannulas aimed at the PFC (3.2 mm anterior and -0.6 mm lateral to bregma and 1.5 mm below dura mater). The lateral ventricle guide cannula was placed -0.9 mm anterior and 1.5 mm lateral to bregma and 3 mm below dura mater [61]. The cannulas were secured to the skull with stainless steel screws and dental cement.

### Effect of food intake on extracellular glucose in PFC

Seven days after surgery, at 4–5 pm and under slight anesthesia, the rats had a concentric custom-made microdialysis probe (effective membrane length: 4.0 mm, 13000 Da cut-off) inserted through the PFC guide cannula and fixed to it with dental cement. Probe *in vitro* glucose recovery was 9 ± 1.82%. Details of probe construction were as previously described [62].

The rats were connected to a swivel system and the probe was attached to a microinjection pump, allowing continuous perfusion with artificial cerebrospinal fluid (145 mM NaCl, 2.2 mM KCl, 1.0 mM MgCl2, 1.2 mM CaCl2, 2.0 mM Na2HPO4, pH 7.4), at a flow rate of 1.5 μl/minute. Overnight perfusion was performed to allow equilibration. Rats were fasted overnight.

On the next morning, the collection of 30-min dialysate samples started at 8 am. Three baseline samples were collected and a known amount of food was offered. The acute food mixture consisted of powdered control or fish-oil diets to which condensed milk was added in the proportion of 1:1.5 (Table 2). The amount of food ingested was measured after thirty minutes, coinciding with the collection of one microdialysate sample. After this 30-min. meal period, food was withdrawn and 7 additional microdialysate samples were collected.

At the end of the experiments, the brains were sliced and photographed for confirmation of membrane location in the PFC.

### Measurement of serum glucose and insulin and adipose tissue mass

The animals were killed by decapitation after an overnight fast and trunk blood was collected and stored at -80°C. Serum glucose was measured enzymatically (glucose oxidase GOD-Trinder, Labtest Diagnóstica, Lagoa Santa, MG, Brazil) and serum insulin was measured by ELISA (Rat/Mouse Insulin ELISA, Millipore). White adipose tissue depots (epididymal, mesenteric and retroperitoneal) were dissected and weighed.

### Measurement of glucose content in microdialysates

Microdialysate samples were immediately stored at -20°C and glucose analysis was performed no later than 24 hours by the method described above, using 10 μl of microdialysate.

### Feeding response to intracerebroventriular glucose and effect of intracortical 2-deoxy-D-glucose (2-DG)

The animals were implanted with two brain guide cannulas, one aimed at the PFC, as described above, and the other aimed at the lateral ventricle (-0.9 mm anterior and 1.5 mm lateral to bregma, and 3 mm below dura mater). The intracerebroventricular (i.c.v.) cannula placement was confirmed by a positive drinking response after administration of angiotensin II (20 ng).

After six days, the rats were fasted from 12:00 to 17:00 h and then received the i.c.v. injection (1 μl in 1 min.) of either vehicle (artificial cerebrospinal fluid) or glucose (20 μg) followed, 30 min. later, by the intracortical injection of either vehicle or 2-DG (100 μg). Immediately after the second injection, a known amount of food was presented. The food consisted of the conventional pelleted control or fish-oil diet the animals received during the 2 months of diet treatment. Ingestion was measured 2 h, 12 h, and 24 h after the injections.

### Statistical analysis

The results are expressed as means ± SEM. Microdialysate glucose levels are expressed as percentage of the mean basal level, obtained by averaging the three baseline samples, collected before food presentation. Dialysate glucose data were analyzed by two-way repeated measures Anova followed by LSD test for multiple comparisons. Food intakes after the injections of vehicle, glucose, or 2-DG were compared by two-way Anova and LSD post-hoc test. The area under the curve relating glucose levels to time was calculated by the trapezoidal rule. Statistical significance was set at p < 0.05. The statistical analyses were performed using the software Statistica 12 (Statsoft, Inc., 2013).

## Abbreviations

2-DG: 2-deoxy-D-glucose; CNS: Central nervous system; i.c.v.: Intracerebroventricular; NPY: Neuropeptide Y; PFC: Prefrontal cortex; POMC: Proopiomelanocortin; PPAR-α: Peroxisome proliferator activator receptor α; PUFA: Polyunsaturated fatty acids; PVH: Paraventricular nucleus of hypothalamus; SREBP-1: Sterol-regulated element binding protein 1; VMH: Ventromedial hypothalamus.

## Competing interests

The authors declare that they have no competing interests.

## Author contributions

IFS contributed to the overall conception and design of the project and carried out the experiments, analysis and interpretation of the data, and preparation of the manuscript. APS and ISA contributed to the overall conception and design of the project and carried out the experiments. VTB contributed with technical support and carried out the experiments. CMON and LMO contributed with critical discussions. MMT contributed to the overall conception and design of the project, analysis and interpretation of the data. EBR conceived the study, and participated in its design and coordination, data analysis and interpretation, and preparation of the manuscript. All authors have read and approved the final manuscript.
